# Addressing a Common Misconception of Simple Additive and Cumulative Drug Inhibition: Multiple Weak Inhibitors of Common Metabolic Pathway Do Not Pose a Strong Interaction Risk!

**DOI:** 10.1002/jcph.70154

**Published:** 2026-01-30

**Authors:** Daniel Scotcher, Avijit Ghosh, Aleksandra Galetin, Amin Rostami‐Hodjegan

**Affiliations:** ^1^ Centre for Applied Pharmacokinetic Research The University of Manchester Manchester UK; ^2^ Independent Consultant; ^3^ Certara Princeton NJ

**Keywords:** drug‐drug interactions, polypharmacy

The global population is becoming older, and prevalence of multimorbidity and polypharmacy is increasing.^1^ Most research into pharmacokinetic drug–drug interactions (DDIs) has focused on pairwise interactions between drugs and corresponding metabolites, while the impact of co‐administration of multiple drugs has received less attention. More recently, the differences in DDI risk between population sub‐groups has received increased attention (e.g., in pediatrics vs adults^2^, ^3^). It is well recognized that simultaneous inhibition of multiple different pathways of a drug's elimination can lead to much larger increases in drug exposure compared with inhibition of only the major pathway.^4^ The potential magnitude of DDI in this scenario is linked with the fraction metabolized (fm) of each pathway for the object (also known as “victim”) drug^5^, ^6^ (Equation 1). In contrast, there is a common misconception about the cumulative effects of multiple precipitant (also known as “perpetrator”) drugs that competitively inhibit the same enzyme. It is often mistakenly perceived that cumulative effects of multiple weak inhibitors are likely to lead to a strong DDI. This misconception arises despite the true relationship being governed by a simple concentration–response relationship, with a well‐known theoretical basis (Equations 1 and 2),^4^, ^5^ analogous to the dose dependence of competitive inhibition. Surprisingly, there is very limited research on multiple inhibitors and their implications on clinical endpoints such as the ratio of area under the curve of the plasma concentration–time profiles (AUCR) in the interaction phase relative to the control,^7^ possibly contributing to such misunderstandings about polypharmacy–DDI risks.

The purpose of this article is to provide clarification on the polypharmacy DDI risk for drugs that inhibit the same metabolic pathway of an object drug. For simplicity, we only consider scenarios involving competitive inhibition, recognizing that practical applications would require consideration of other mechanisms of DDI such as noncompetitive inhibition, irreversible inhibition (inactivation), and induction. The scenarios investigated here also apply to potential changes in exposure of an inhibitor that may arise due to inter‐patient variability (e.g., organ impairment), or due to a polypharmacy‐related DDI affecting exposure of the inhibitor itself.

(1) $$\mathrm{AUCR}\;=\frac{1}{\sum_{j=1}^{n}\left(\frac{fm_{j}}{1+\sum_{k=1}^{p}\frac{\left[I_{k}\right]}{Ki_{k,j}}}\right)+\left(1-\sum_{j=1}^{n}fm_{j}\right)}\;$$

(2) $$\mathrm{AUCR}\;=\frac{\mathrm{AU}C_{\mathrm{inhibited}}}{\mathrm{AU}C_{\mathrm{control}}}\;$$

where AUC_control_ and AUC_inhibited_ are the AUCs for the object drug when administered alone, and co‐administered with p other precipitant/inhibitor drugs, respectively. fm_j_, [I_k_], and Ki_k,j_, are the fraction metabolized by the j‐th metabolic pathway for the object drug, concentration for the k‐th inhibitor drug, and the inhibition constant for the k‐th inhibitor drug, respectively.

The ICH M12 Drug Interaction Studies Guidance defines weak, moderate, and strong CYP inhibitors as causing ≥1.25‐ to <2‐fold, ≥2‐ to <5‐fold, and ≥5‐fold increase in AUC of a sensitive index CYP substrate.^8^ Assuming a sensitive CYP substrate will have fm ∼ 1, the corresponding [I]/Ki for weak, moderate, and strong CYP inhibitors will be ≥0.25 to <1, ≥1 to <4, and ≥4, respectively. Hence a 4‐fold difference in [I]/Ki (after correction for exposure) may be a useful approach to benchmark between weak, moderate, and strong inhibitors.

When fm = 1 the relationship between ∑[I]/Ki and AUCR is linear (AUCR = ∑[I]/Ki + 1, Figure 1a). However, this relationship is not proportional due to the non‐zero intercept.^5^, ^6^, ^9^ Particularly at low ∑[I]/Ki values (e.g., from multiple weak inhibitors), the relative change in AUCR will be much lower than the relative change in [I]/Ki (Figure 1b). In other words, to see a change in AUCR from 1.25 to 2 for a weak inhibitor, the co‐administration of three additional inhibitors with equivalent [I]/Ki would be required, or a 4‐fold increase in the dose or exposure of a single inhibitor.

**Figure 1:**
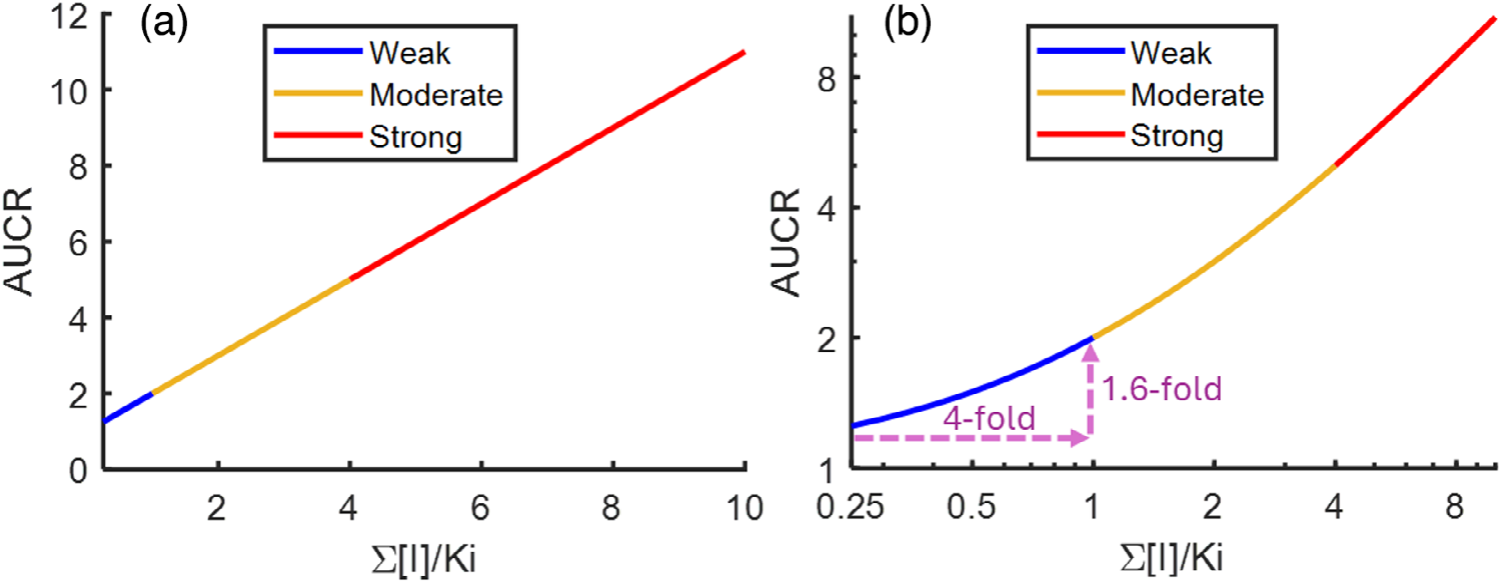
Relationship between ∑[I]/Ki and AUCR expected for competitive inhibitors of object drug that has elimination by one metabolic pathway (i.e., fraction metabolized = 1), following Equation (1). (a) The relationship with linear‐scale axes and (b) the relationship with log‐scale axes, and highlights the changes in ∑[I]/Ki and AUCR for weak inhibitors (i.e., ∑[I]/Ki from 0.25 to 1). Figure created using Matlab R2023a (The MathWorks Inc., Natick, MA).

In reality, DDI risk analyses will initially consider the inhibitor with the strongest in vivo inhibition potential (i.e., the highest [I]/Ki), with the effects of any further inhibitor drugs in a polypharmacy setting being considered relative to that strong inhibitor. As shown in Table 1, the overall inhibition of an enzyme achieved by addition of series of exponentially weaker inhibitors reaches a limit defined by the relative inhibition potential of the inhibitor. As such, a maximal polypharmacy effect of 2‐fold is expected in the case where each next inhibitor has half the exposure/potency ([I]/Ki) ratio of the last‐added inhibitor. Thus, cumulative effects of multiple inhibitors against the same enzyme will only likely be clinically relevant if the inhibitors have similar [I]/Ki.

**Table 1:** Overall Inhibition^a^ of an Enzyme in a Polypharmacy Set Up with N Inhibitors Under Different Scenarios Where the n‐th Inhibitor Has Lower Relative Inhibition Potential ([I]/Ki) Than the n − 1 Inhibitor

	Relative [I]/Ki of Each Next Inhibitor Added [I_n_]/Ki_n_ = x * [I_n − 1_]/Ki_n − 1_				
N inhibitors	x = 1	x = 0.9	x = 0.75	x = 0.5	x = 0.25
**1**	1	1.00	1.00	1.00	1.00
**2**	2	1.90	1.75	1.50	1.25
**3**	3	2.71	2.31	1.75	1.31
**4**	4	3.44	2.73	1.88	1.33
**5**	5	4.10	3.05	1.94	1.33
**∞ [1/(1 − x)]** ^b^	∞	10	4	2	1.33

^a^ Overall inhibition = $\sum_{n\;=1}^{N}\frac{\left[I_{n}\right]}{Ki_{n}}$.

^b^ Convergence of the geometric infinite series, where x is the relative [I]/Ki of each next inhibitor added.

Further, most marketed drugs have multiple routes of elimination, such that the fraction metabolized for the major enzymatic pathway is commonly less than 1. The sensitivity of AUCR to increase with inhibitor dose or with polypharmacy is reduced with lower fm. Interactions involving a substrate with fm > 0.7 may be of potential concern in the context of clinical polypharmacy effects (e.g., AUCR increasing by ∼50% with multiple inhibitors vs one inhibitor; Figure 2, pink dashed boxes). A 2‐fold increase in ∑[I]/Ki leads to a modest increase in AUCR of ∼50% relative to the single inhibitor scenario, but this occurs only for substrates with very high fm (>0.9). However, few clinically used drugs have such high fm values. Another plausible but even less likely scenario that involves 4‐ or 5‐fold increases in ∑[I]/Ki (i.e., more than four drugs that inhibit an enzyme with similar inhibition potential, Table 1) could lead to increase in AUCR of >2‐fold compared with AUCR for the inhibitor with highest [I]/Ki (Figure 2, red dashed boxes). These theoretical changes in AUCR are consistent with reported dose‐dependent DDIs.^7^

**Figure 2:**
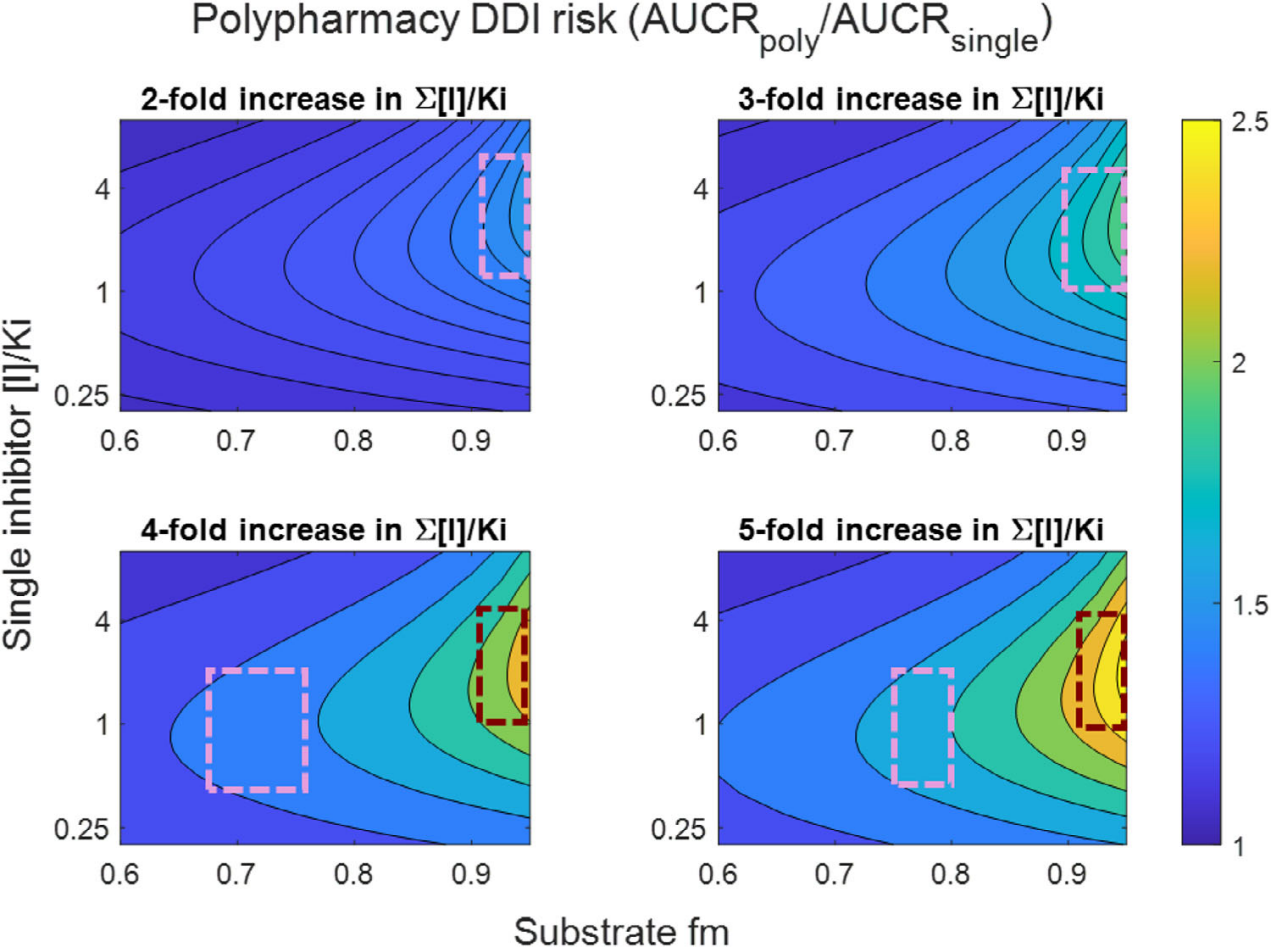
Simulated impact on drug‐–drug interaction (DDI) risk for various scenarios of polypharmacy using the mechanistic static model (Equation 1). The mechanistic static model here provides a useful tool to illustrate the key factors and underlying relationships that govern DDIs through competitive inhibition, despite known limitations.^10^, ^11^ Polypharmacy effect (represented by color of plot) is calculated based on the fold difference in AUC ratio (AUCR) for different relative increases in overall inhibition (∑[I]/Ki) against a base scenario (single inhibitor). Dark blue represents no difference between AUCR for polypharmacy and AUCR for single inhibitor, with warmer colors representing increasing effects of polypharmacy on AUCR. Scenarios consider a range of fm for the substrate, and varying inhibition potential ([I]/Ki) for the base scenario of a DDI involving a single inhibitor. Boxes with pink and red dashed lines represent selected scenarios where fm > 0.7 or fm > 0.9, respectively, as discussed in the text. Figure created using Matlab R2023a (The MathWorks Inc., Natick, MA).

In conclusion, polypharmacy DDI risk for drugs that inhibit the same metabolic pathway for an object drug is typically of low concern, as previously suggested.^4^ The current analysis using a mechanistic static model has revealed the edge cases where additive effects of co‐administration of multiple competitive inhibitors may cause clinical DDIs that have higher AUCR than those observed when only co‐administering the inhibitor with highest [I]/Ki. Therefore, risk assessment for DDIs in polypharmacy scenarios should consider the properties of the object drug and each precipitant drug in a mechanistic manner. Preferably, physiologically based pharmacokinetic modeling should be applied to recognize time‐varying interaction effects of each drug at different sites, interindividual variability in exposure, and the possibility that some drugs may act simultaneously as object and precipitants of DDI.

## Conflicts of Interest

The authors declare no conflicts of interest.

## Funding

No specific funding was received for this work.

## Data Sharing

Matlab code to create the figures in this article are publicly available through the FigShare repository: https://doi.org/10.48420/29246777
